# Youth participation coach learning: A scoping review

**DOI:** 10.1371/journal.pone.0356171

**Published:** 2026-08-18

**Authors:** Li Quan Warrick Tan, James Barkell, Donna O’Connor

**Affiliations:** Sydney School of Education and Social Work, Faculty of Arts and Social Sciences, The University of Sydney, Sydney, Australia; Portugal Football School, Portuguese Football Federation, PORTUGAL

## Abstract

Coaching in the youth participation sport domain has garnered considerable attention amongst researchers, sport development organisations and coach education bodies over the last decade. However, little is empirically known of how youth participation sport coaches learn and how to support them in their development of coaching skills and knowledge. A scoping review was therefore conducted with the aim of synthesizing the research that analysed the perspectives and experiences of youth participation coaches, and to suggest future research agendas. Informed by Arksey and O'Malley's five-stage framework (2005), leading sports and education databases were searched for empirical literature on coach education and learning in youth participation sport published between January 2010 to April 2025. Following the screening stages, eleven articles were descriptively mapped. To unveil further insights, quantitative and qualitative analysis was conducted. Quantitatively, the majority of the articles were focussed on examining the effectiveness of coach education programs related to autonomy-supportive coaching in team sports and were conducted in western countries. The qualitative analysis revealed four key topics: learning enablers, learning challenges, coach learning outcomes, and factors influencing the impact on coaches’ practice. Based on the review, we propose recommendations for future research (i.e., examining impact on coaches’ practice, studies in non-western contexts, research across different sport types, studies employing larger and more diverse samples of coaches, and explorations of learning in relation to the coaches’ role within specific contexts).

## Introduction

Coaching in the youth participation sport domain is characterised by sessions that are focused on improving athlete health, well-being and holistic growth [1,2]. The concept of a ‘youth participation coach’ lacks clear definition, often overlapping with terms like ‘recreational’ [2], ‘grassroots’ [1], or ‘community’ [3] coaches, all of whom similarly aim to promote mass participation and positive early sporting experiences for their athletes. Drawing on Lyle and Cushion [1], these coaches generally operate within the participation domain of recreational sport, prioritising enjoyment, basic skill development, and low intensity sporting engagement [2]. Similarly, grassroots coaching emphasises mass participation and sport initiation [1], while community sport [3] aims to foster social interaction among diverse groups through sport. Despite nuances between these contexts (recreational, grassroots, community), they share core aims and characteristics of participation-focused coaching [1].

As architects of learning environments, coaches can foster the holistic and healthy development of their youth athletes [4,5]. To coach effectively in the youth participation domain, research highlights the importance of coaches intentionally planning developmentally appropriate sessions to enhance athletes’ skill development (e.g., fundamental movement skills [6]), develop a range of desirable athlete outcomes (e.g., competence, confidence, connection and character [7]), and influence long-term sporting outcomes (e.g., participation, performance, personal development [8]). Additionally, Côté and Abernethy [5] argued from a psychosocial perspective, that youth athletes should be developed according to three phases: sampling years, age 6–12; specialising years, age 13–15; investment years, age 16 and above. Côté and Abernethy [5] noted that recreational participation should be the focus in the sampling years, while athletes may choose to pursue sport at either a recreational or a performance level in the specialising years. Designing and delivering effective sessions that are developmentally appropriate for their athletes therefore necessitates coaches possessing specialised coaching knowledge [9]. While research has explored the specific coaching knowledge required for youth participation coaches to coach effectively, a gap remains in understanding how these coaches acquire and implement that knowledge [10].

Coaches learn from a range of experiences, which may be classified within a continuum of formal and informal learning sources based on how highly structured the course or workshop is designed [11]. Research has explored the perceived value coaches place on these different sources of learning [12–15]. A study with youth sport coaches indicated a preference for informal learning, as it aligns closely with their practical experiences and individual learning needs [13]. Social learning activities like mentoring and learning communities are also highly valued, facilitating learning through discussion of issues directly relevant to their own coaching practice [14]. Similarly, interaction with coaching peers is consistently identified as a preferred source of knowledge [12,15]. While informal learning often holds greater perceived value, less experienced coaches tend to appreciate formal sources for providing foundational knowledge [16] or when the content is directly applicable to their specific sport [13]. A notable trend, arguably catalysed by the COVID-19 pandemic, is the increasing adoption of online and hybrid modes of learning, which as a result, improved accessibility of learning for coaches [17,18]. Along this line of inquiry, Cushion and Townsend’s [19] review called for more empirical evidence to validate technology-enhanced learning in coach education. Consequently, research has indicated that the perceived value of learning may be dependent on its delivery [20,21] and alignment with the coach’s needs [22,23]. While previous work has examined how coaches learn [16,24,25], limited research addresses how youth participation coaches can be effectively prepared for their unique role(s) [26].

One strategy to address the challenge of supporting coaches in improving their practice is through the provision of coach education programs (CEPs [2,27]). Particular to youth participation coaches, their education and training needs of youth participation coaches can differ significantly from those of coaches in other contexts [28]. This is in part, due to the unique (and sometimes multiple) roles they are responsible to enact [26,29]. To ensure appropriate capacity building [28] of these coaches, the content of such CEPs may include coaching pedagogy [30], holistic youth development [8], as well as health, safety, and protection of youth [31]. However, attendance at such CEPs can be a challenge for these coaches who largely coach on a part-time or voluntary basis [1,32], who may perceive committing time to attend and pay for CEP as a burden [33–35] and of little benefit [36]. This is further problematised as coach education for these coaches does not adequately support the “complex coaching context” [37 p. 267] in which they operate in.

Recent reviews of the literature revealed some findings relevant to youth participation coach education and learning [10,21,38]. For example, Walker et al. [10] recommend future research should explore coaches’ learning motivations and barriers and the impact of their learning on outcomes such as coach retention and athlete skill development, within recreational and developmental contexts. Further, Li et al. [38] emphasised that community coaches face greater challenges in improving their practice due to limited access to quality resources and development opportunities. Despite these insights, existing reviews have yet to synthesize research specifically focused on the perceptions and experiences of youth participation coaches.

To address this gap, a comprehensive scoping review of youth participation coach education and learning literature is essential to map current research and identify key developments. This review will examine study designs (e.g., cross-sectional or longitudinal, quantitative or qualitative), theoretical frameworks, sport types (team, individual) and coaching curriculum and delivery strategies). This analysis will inform the identification of research gaps and guide future research priorities. Therefore, the aim of the study is to conduct a scoping review of current coach education and learning literature with a specific focus on the perspectives of youth participation coaches.

## Methodology

### Overview of scoping review methodology

The scoping review methodology was chosen as it allowed the researchers to achieve three primary aims [39,40]. Firstly, the scoping review aims to map a broad and diverse body of literature on youth participation coach education and learning. Secondly, scoping reviews are designed to include a variety of literature, including qualitative, quantitative, and mixed-methods research. Thirdly, scoping reviews allow for a descriptive overview of the current body of literature without critically appraising the literature. Thus, other types of reviews such as a systematic review were not deemed appropriate and did not align with the researchers’ aims. Unlike a systematic review, the scoping review methodology does not seek to synthesize evidence or aggregate findings, nor does this methodology aim to assess the quality of evidence of the included studies [41–43]. In other words, rather than critically analysing the evidence of included studies and presenting the results on a micro level, the purpose of a scoping review is to provide insights into overall trends at a more macro level, and this is done by collating, summarizing, and reporting of results [39,44]. The eventual outcome of a scoping review can allow a more thorough investigation of emerging evidence which subsequently, facilitate identifying and analysing research gaps. This strategy is appropriate to scope the broad and diverse youth participation coach education and learning literature [40]. The scoping review followed a five-stage process: (a) identifying the research question; (b) identifying relevant studies; (c) study selection, (d) charting the data; and (e) collating, summarising, and reporting the results [39]. The Preferred Reporting Items for Systematic Reviews and Meta-Analyses extension for Scoping Reviews (PRISMA-ScR) checklist [45] was used to guide the reporting of this review (refer to S1 File). The study protocol for this scoping review was registered in the Open Science Framework (registration link: https://osf.io/8t9us). Details regarding the steps taken for each stage are described in the following sections.

### Identifying the research question

In line with scoping review recommendations [46], we used broad research questions combined with clear definitions of the concepts relevant to the study’s scope. An overarching research question guided the scoping review: What do we know about youth participation coaches’ perceptions of learning and experiences learning from various sources?

### Identifying relevant studies

To ensure a comprehensive and interdisciplinary review, we searched six major electronic databases covering coaching, medicine, organizational science, pedagogy, psychology, and sociology to identify studies addressing sport from a range of theoretical perspectives. The databases included were: SPORTDiscus, ERIC, Scopus, Web of Science, APA PsycINFO, and Medline, using a search combination of relevant keywords: volunteer OR participation OR grassroots OR recreation* AND youth OR adolescen* OR teen* OR child* OR “elementary school students” OR “primary school students” AND coach* OR practitioner AND learn* OR education OR development AND sport. The literature search was conducted up to April 28, 2025.

Prior to screening, the research team discussed and provided an inclusion and exclusion criteria to guide the article screening process. The specific inclusion and exclusion criteria are illustrated in Table 1.

**Table 1:** Inclusion and exclusion criteria.

Inclusion	Exclusion
Language: English	Language: Non-English
Publication type: Peer reviewed journal	Publication type: Grey and non-peer reviewed literature
Date range: January 2010 to April 2025	Date range: 2009 and before
Study participants: Coaches who are the main participants of the study or the researcher was able to separate the coaches’ results from the other participants	Study participants: Coach developers, sport administrators, athletes, parents, physical education teachers, primary teachers, early childhood teachers
Coaches’ profile/context or coaching setting: Participation, community, recreational, volunteer, grassroots	Coaches’ profile/context or coaching setting: Performance, elite, disability, parasport
Coaches’ athletes: Children, youth (15 years of age and below)	Coaches’ athletes: Youth (16- to 18-year-olds), adult (18 years and above)
Outcomes focused on: Coaches’ perceptions (views, attitudes) of or experiences of coach learning (e.g., perceptions / experiences of coach education programs)	Outcomes focused on: Coaching practice (e.g., pedagogy); coach development (e.g., pathways, trajectory); coaching beliefs (e.g., philosophy)
Study type: Quantitative and/or Qualitative	

### Study selection

**Fig 1:**
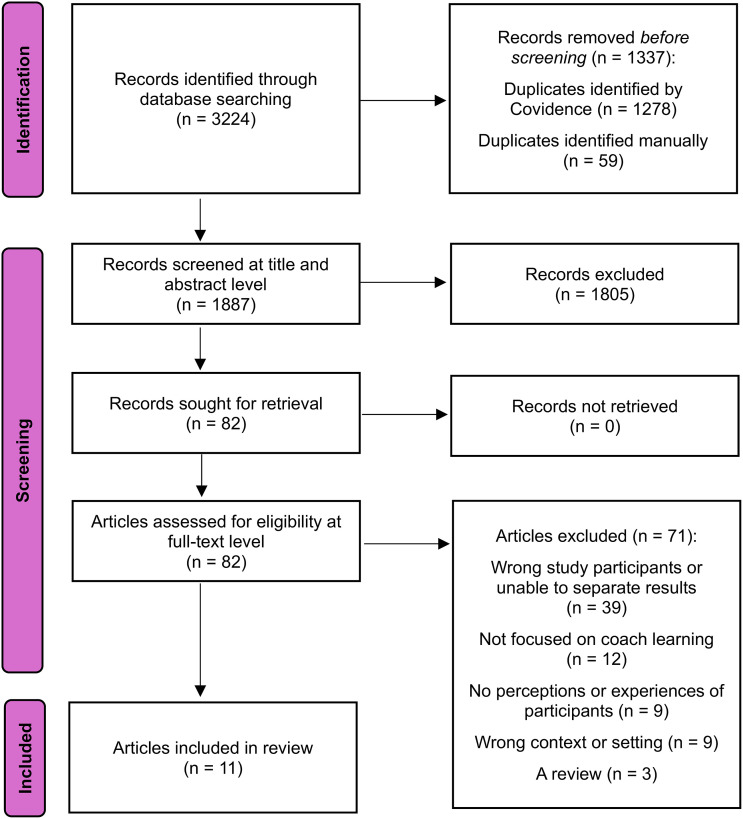
Preferred Reporting Items for Systematic reviews and Meta-Analyses flowchart.

After completing the initial electronic database search, all selected references (n = 3224) were imported to Covidence [47], a web-based systematic review software that facilitated the review process [48]. Duplicates were removed and the remaining records were screened according to the PRISMA-ScR guidelines. The PRISMA flowchart (Fig 1) shows the number of records found and screened in each step of the scoping search described. Across the six databases, 3224 results were returned, of which 1337 were duplicate records. The research team screened the title and abstract of each of the remaining 1887 studies followed by the screening of 82 full-text studies, of which 11 studies were included for further analysis. Throughout the screening process, two reviewers separately screened each study for eligibility. In the event of any discrepancies in decisions (e.g., to include or exclude study) between the two reviewers at each stage of the screening, they were first resolved through discussion among the original reviewers. If the two original reviewers did not reach consensus, a third reviewer was consulted to make the final decision.

### Charting the data

All sources identified as relevant for inclusion were listed on a Microsoft Excel spreadsheet. These articles were then read in full, and data were charted on the Excel spreadsheet by the first author with the following categories: author(s), year of publication, aim, study design, method of data collection, method of analysis, theoretical construct, learning source, learning mode, course / program type, course / program duration, course / program name, participants, and key findings (Table 2). The data charting spreadsheet was shared and discussed among the author team.

**Table 2:** Characteristics of included studies.

General study details			Coach Participants				Methodology				CEP Characteristics					Key findings
Author(year)	Country of study	Sport type	Gender (*N*)	Coaches’ experience	Prior coaching qualifications (*N*)	Coaches’ Status	Study aim	Study design	Data collection	Theoreticalconstruct	Learning source(mode)	Name of CEP	CEP scale (duration)	Key content / topics	Key delivery / strategies	
Langdon et al. (2015) [49]	USA	Team:Football	M = 5; F = 2	Mean = 6.43 yrs (SD = 6.44)(Intermediate)	NS	V	To evaluate the prevalence and implementation of a training emphasizing the use of autonomy supportive coaching behaviours among youth soccer coaches in game-play situations as well as evaluating its effects on motivational processes among athletes.	LONG: Mixed-methods explanatory research	QUAN: Athlete questionnaire; Coaching behaviours*QUAL: Coaches’ reflections (during CEP)	Self-determination theory	Formal(Both)	“Autonomy-supportive coaching training”	SM (12 weeks)	Autonomy-supportive coaching	Written and verbal feedback by coach developer; supplementary online learning; systematic observation of coaching behaviours; self-reflection	Coaches were not able to significantly modify their coaching behaviours in game play yet reflectively reported modest implementation of autonomy supportive behaviours. Coaching experience influenced the variety of autonomy supportive coaching behaviours demonstrated. Coaches believed the training influenced their coaching style/philosophy in relation to coach-athlete relationship and communication styles, emphasizing choice and rationales.
Larsen et al. (2015) [50]	FranceNorway	Team: Football	FRA: M = 5; F = 1NOR: M = 10; F = 2	FRA: Mean = 5.67 yrs (SD = 1.63)NOR: Mean = 6.92 yrs (SD = 5.26)(Intermediate)	Yes = 9; No = 9	V	To examine how grassroots coaches in youth football perceive their coaching practices after participating in a community-based coach education program aimed at optimizing their experiences in youth sport, namely the Empowering Coaching Training Program, based on self-determination theory and achievement goal theory.	CS: Qualitative exploratory research	QUAL: 1−1 interview (post-CEP)	Self-determination theory; achievement goal theory	Formal(Both)	Empowering Coaching Training Program	SM (1 day)	Autonomy-supportive coaching	Coach developer-facilitated workshop; supplementary workbook and online learning	All coaches had a shared understanding of the philosophy of the CEP, and to apply several of the strategies they had learnt during the workshop. The coaches perceived that the CEP supported their efforts to develop and implement strategies to stimulate intrinsic motivation, enjoyment and long-term participation among the players. There were some differences between coaches from France and Norway (e.g., rules and involvement), but the similarities were more evident, supporting the universality of applying self-determination theory in the youth sport setting.
Strachan et al. (2016) [51]	Canada	Team: Football, Hockey, Volleyball	M = 3; F = 1	4 to 12 yrs (*N* = 4)(Mixed)	NS	NS	To design and deliver a technology-based Positive Youth Development program (Project SCORE!) and explore its impact on coaches.	CS: Qualitative exploratory research	QUAL: 1−1 interview (post-CEP)	Positive youth development; ecological theory	Informal(Online)	Project SCORE!	SM (6–12 weeks)	Holistic youth coaching (Positive Youth Development)	Self-paced online learning	Four themes (and sub-themes) were reported in the pilot implementation: (a) program organization (preparation, delivery of lessons); (b) positive aspects about the program (website, lessons/sessions, personal growth, parent reaction); (c) challenges about the program (participant involvement, time away from training); and (d) suggestions for change (mix and match lessons, applicability to younger ages).
Søvik et al. (2017) [52]	Norway	Team: Football	QUEST: M = 102; F = 11 INT: M = 10; F = 2	QUEST: Mean = 7.01 yrs (SD = 4.82)INT: Mean = 6.92 yrs (SD = 5.26)(Intermediate)	QUEST: NSINT: Yes = 5; No = 7	NS	To explore grassroots coaches’ perceptions of the content of a one-day coach education workshop (Empowering Coaching Training Program), the program’s applicability, their use of the content, and the perceived barriers to implementing the program in their coaching practice.	CS: Convergent parallel mixed-methods exploratory research	QUAN: Questionnaire (post-CEP)QUAL: 1−1 interview (post-CEP)	Effective program implementation	Formal(Both)	Empowering Coaching Training Program	SM (1 day)	Autonomy-supportive coaching	Coach developer-facilitated workshop; supplementary online learning	The quantitative results indicate that the coaches were mainly positive about the CEP content and found it easy to apply and adapt to. Few coaches seemed to apply the program principles to a great extent. Qualitatively, coaches reported barriers related to program characteristics (i.e., lack of follow-up); individual factors (i.e., lack of time; players’ time); and organizational factors (i.e., lack of support from club leadership or club officials, lack of a shared understanding of the program with their co-coaches).
Solstad et al. (2018) [53]	Norway	Team: Football	QUEST: M = 174; F = 19**INT: M = 10; F = 2	QUEST: Mean = 4.26 yrs (SD = 2.10)**INT: Mean = 6.92 yrs (SD = 5.26)(Intermediate)	QUEST: Yes = 106; UN = 87**INT: NS	NS	To investigate differences in self-reported behaviours among youth football coaches from pre- to post-season, and additionally, their evaluative reactions to participation in the Norwegian arm of the Empowering Coaching Training Program.	LONG: Sequential mixed-methods explanatory research	QUAN: Questionnaire (pre- and post-season)**QUAL: 1−1 interview (post-season)	Self-determination theory; achievement goal theory	Formal(Both)	Empowering Coaching Training Program	SM (1 day)	Autonomy-supportive coaching	Coach developer-facilitated workshop; supplementary coaching manual and online learning	Coaches’ empowering and disempowering behaviours did not differ from pre- to postseason. Qualitatively, the post-season interviews showed that participation in the CEP led coaches to reflect on their coaching practices, facilitating an increased focus on enabling autonomy and involvement for the athletes and more attention paid to the athletes’ feelings of mastery.
Cope et al. (2021) [54]	England	Mixed	M = 1; F = 2	3 to 6 yrs (*N* = 3)(Intermediate)	Yes = 3	Mixed	To investigate the impact a theoretically informed learning programme had on coaches’ ownership of, and feelings towards, being engaged in their learning, and how this impacted their subsequent practice.	LONG: Mixed-methods explanatory research	QUAN: Coaching observation (during CEP)*QUAL: 1−1 interview (pre- and post-CEP); reflective conversations (during CEP)	Critical pedagogy; collaborative action research	Formal(Both)	“Freirean-informed coach education program”	SM (9–13 weeks)	Coaching practice; Coach learning	Online resources (blogs, podcasts and videos); coach developer-facilitated reflective conversations; systematic coaching observations	Based on Freire's critical pedagogy, three themes were identified based on analysis: (a) freedom to learn, (b) feeling cared for, and (c) becoming (or not becoming) more reflexive. Quantitative data highlighted where this led to changes in the coaches’ behaviours.
Barrero et al. (2022) [55]	Spain	Team: Football	M = 140; F = 13	4 to 7 yrs (*N* = 61)7–10 yrs (*N* = 44)> 10 yrs (*N* = 14)(Mixed)	Yes (Level 2) = 71; Yes (Level 3) = 60Some NS	NS	To identify the profile and education of grassroots football coaches (working with 8- to 12-year-olds) of Spanish professional clubs (first and second male division, and first female division), as well as the qualities and characteristics they should have.	CS: Quantitative exploratory research	QUAN: Questionnaire	NS	NA	NA	NA	NA	NA	Coaches considered it very important to have pedagogical skills and to use coaching methods that focus on learning rather than on results. Coach participants considered that the training received in the official coaching courses did not adequately qualify them to carry out the sporting and human education of boys and girls in the under-10 and under-12 age groups. The coaches also felt that the contents of the coaching course should be adjusted to the requirements of these age groups.
O’Brien et al. (2022) [56]	Ireland	Team: Gaelic Football	M = 5; F = 3	2 to 20 yrs (*N* = 5)UN (*N* = 3)(Mixed)	Yes = 6; UN = 2	V	To examine Irish volunteer coaches’ experiences of the content and delivery of the Gaelic4Teens coach education initiative and further seeks to evaluate if participants coaching behaviour changed as a result.	LONG: Qualitative explanatory research	QUAL: Focus-group interview (pre- and post-CEP)	NS	Formal(Both)	Gaelic4Teens Program	SM (16 weeks)	Autonomy-supportive coaching and National Sport Federation guidelines	Face-to-face session; live webinars; self-paced online learning; class discussion via e-learning platform	Overall, the CEP had a meaningful impact on coaches’ abilities to competently work with female adolescents. Three overarching themes were: (a) coach education: coach appreciation of online and blended learning approaches; (b) coach environment: the coach’s provision of player autonomy, conﬁdence and competence; and (c) coach behaviour: holistic coaching philosophy, using robust coaching pedagogies in practice.
Barnes and Curtner-Smith (2023) [57]	USA	Team: Football	M = 2	6 yrs and 10 yrs(Intermediate)	No = 2	V	To describe the impact of a progressive coach education program on two grassroots youth soccer coaches’ perspectives and practices, and the factors that helped and hindered the CEP’s effectiveness.	LONG: Mixed-methods descriptive case study	QUAN: Coaching observation (pre- and post-CEP)*QUAL: 1−1 interview (pre-, during, and post-CEP); coach-created materials (pre- and post-CEP)	Occupational socialization theory	Formal(Both)	United States Soccer Federation Grassroots Coaching License	LG (1 day)	Coaching pedagogy (Game-based coaching)	Introductory online module; coach developer-facilitated classroom discussion; session planning with on-field coaching; systematic observations of coaching behaviours	The CEP had a significant impact on one coach but negligible on the other coach. The two coaches’ occupational socialization helped explain these differential effects. The study suggests that CEPs should have a greater impact on coaches if they are relatively lengthy, include follow-up support, and coach developers are aware of coaches’ acculturation and organizational socialization
Barnes and Curtner-Smith (2024) [58]	USA	Team: Football	M = 1	About 3 yrs(Novice)	No = 1	V	To describe how one grassroots youth soccer coach was influenced by a progressive coach education programme, and the factors that supported or negated the CEP’s effectiveness.	LONG: Mixed-methods descriptive case study	QUAN: Coaching observation (pre- and post-CEP)*QUAL: 1−1 interview (pre-, during, and post-CEP); coach-created materials (pre- and post-CEP)	Occupational socialization theory	Formal(Both)	United States Soccer Federation Grassroots Coaching License	LG (1 day)	Coaching pedagogy (Game-based coaching)	Introductory online module; coach developer-facilitated classroom discussion; session planning with on-field coaching; systematic observations of coaching behaviours	The CEP had little influence on the coach's beliefs and pedagogies. The coach’s professional socialization was not powerful enough to overcome his acculturation or his organizational socialization. The study indicates the need for lengthy CEPs, follow-up support and suggests coach developers should deconstruct their coaches’ acculturation and mediate any negative organizational socialization to which coaches may be subjected.
Swartz et al. (2025) [59]	USA	Mixed	P1: M = 17; F = 16P2: M = 18; F = 24	P1: Mean = 4.72 yrs (SD = 5.47)P2: Mean = 6.14 yrs (SD = 4.44)(Intermediate)	NS	V	To explore the impact of the Youth Sport Coach Fellowship Program which was aimed at empowering grassroots coaches with the knowledge, resources, and networks needed to promote holistic athlete development and foster positive youth outcomes both on and off the field.	LONG: Mixed-methods exploratory action research	MIXED: Written and video assignments, discussion posts, reflections (during CEP); survey (post-CEP)	Adult learning theory	Formal(Online)	Youth Sport Coach Fellowship Program	SM(Phase 1: 26 weeks,Phase 2: 14 weeks)	Holistic youth coaching	Online synchronous learning (e.g., live class discussion, coach developer-led video calls) and asynchronous learning (e.g., self-paced learning modules, capstone project)	Overall, the action research approach supported exploring the design of the CEP in terms of its effectiveness on coaches. Four key areas in relation to the study purpose were: (a) combating coaching isolation; (b) supporting coaches’ desire to learning; (c) harness the power of cross-sport connections; and (d) importance of well-prepared facilitation.

*Only qualitative data from this study was included in the qualitative analysis as the data met the inclusion criteria (i.e., measured coaches’ perceptions).

**Only the qualitative data from this study was included in the qualitative analysis as the qualitative study sample met the inclusion criteria (i.e., coaches’ athletes are 15 years-old and below).

Note. M = male; F = female; FRA = France; NOR = Norway; P1 = Phase 1; P2 = Phase 2; UN = Unknown; NS = Not stated in study; UEFA = Union of European Football Associations; FT = Full-time; PT = Part-time; V = Volunteer; QUEST: Questionnaire; INT: Interview; CS = Cross-sectional; LONG = Longitudinal; QUAN = Quantitative; QUAL = Qualitative; MIXED = Mixed-methods; NA = Not Applicable; SM = Small-scale; LG = Large-scale.

### Data analysis

Descriptive statistics was conducted on the selected articles for study context (e.g., geographical location of study, sport type), participant characteristics (e.g., gender, coaches’ experience) and CEP characteristics (e.g., source of learning, mode of learning). The qualitative data within each article were analysed using Peters et al.’s [60] guidelines for scoping reviews and Braun and Clarke’s [61] approach to topic summaries. To categorise, map, and summarise the diverse results and insights from the studies, the analysis was informed by the researcher’s understanding of the key concepts in the coach education and learning literature. In synthesizing the content from the selected articles, the researcher (i.e., first author) followed a six-step process. First, the researcher familiarized himself with the eleven articles by reading and re-reading the contents of the articles while making notes on interesting findings. Next, preliminary codes (e.g., ‘access to coaching resources’) were written based on deductively identifying general statements that address the research questions of the review and are evident across sections of the articles. Third, related codes were clustered together, which led to the discovery of potential topics and subtopics. For example, the codes ‘access to coaching resources’ and ‘access to online platforms’ were clustered as ‘accessibility’. Fourth, preliminary topics and subtopics were reviewed by splitting, merging, or discarding irrelevant topics and this was subsequently incorporated into an overall topic summary. Fifth, in refining topics, clear definitions were provided to ensure the names given to the topics (e.g., ‘learning enablers’) were reflective of the relationships between topics (and subtopics). Finally, the results were written in a manner that adequately explains and summarises the analysed content. Both the extracted data from the eleven articles (i.e., in tabular form) and topic summary (i.e., in diagrammatic form with descriptive findings) were shared with the research team, where multiple rounds of discussion, critique and validation were conducted. In the following section, we present a descriptive summary and a detailed topic summary of the studies identified in this review.

## Results

### Descriptive summary

To provide an overview of the youth participation coach education and learning literature, we analysed the contents of the eleven studies and descriptively mapped the results (Table 2).

Descriptive statistics were computed for some of these categories of contents (e.g., study context, participant characteristics) and this is illustrated in S1 Table.

Geographically, the eleven studies were conducted in Europe (six studies, 54.5%) or North America (five studies, 45.5%), with all conducted in western countries. Regarding the sport investigated, team sports were the most prevalent focus (nine studies, 81.8%), particularly football (seven studies, 63.3%). Most studies involved coaches working with both male and female participants (nine studies, 81.8%) and slightly over half specifically examined the views of volunteer coaches (six studies, 54.5%). Coaches with between 3–10 years of coaching experience were the most common participants (seven studies, 63.6%), although some research focused on novice coaches (one study, 9.1%) or included a mix of coaching experience levels (three studies, 27.3%). Prior coaching qualification were reported in seven studies: one exclusively investigated qualified coaches (9.1%); two examined non-qualified coaches (18.2%); and four included a mix of coaches with and without coaching qualifications (36.4%). In the remaining four studies (36.4%) prior coaching qualification were not reported at the time of participation.

Descriptive statistics were also computed for the eleven studies based on CEP characteristics (see S2 Table). Ten of the eleven studies (90.9%) reported coaches’ perspectives on different CEPs, each centred on specific goals or coaching principles (e.g., autonomy-supportive coaching, holistic youth coaching). The nine studies examining these CEPs employed a formal structure, delivered either fully online (two studies, 18.2%) or in a blended format (eight studies, 72.7%). Most of these CEPs were small-scale initiatives (eight studies, 72.7%). The duration of the CEPs varied, ranging from less than one week (five studies, 45.5%) to over twelve weeks (three studies, 27.3%), with two studies (18.2%) lasting between one and twelve weeks. The key content areas explored in these studies included autonomy-supportive coaching (five studies, 45.5%), coaching pedagogy (two studies, 18.2%), holistic youth coaching (two studies, 18.2%), and other coaching-related topics (one study, 9.1%).

The majority of studies employed mixed methods (seven studies, 63.6%) approaches, often aiming to integrate quantitative measures of coach behaviour to enrich and validate the qualitative findings (e.g., [50,54,57,58]). Seven studies adopted a longitudinal design (63.6%) while the remaining four studies (36.4%) used a cross-sectional design. Regarding research designs, a fair distribution of exploratory (five studies, 45.5%), explanatory (four studies, 36.4%), and descriptive (two studies, 18.2%) approaches were observed. In terms of theoretical frameworks, while two studies did not explicitly state a guiding theory [55,56], the remaining studies drew upon diverse perspectives (e.g., self-determination theory, occupational socialization theory) and in some cases, integrated multiple theoretical lenses (e.g., positive youth development and ecological theory [51]). Most of the analyses (four studies, 36.4%) focused on the content delivered within the CEP. Specifically, three studies grounded their analysis in motivational theories [49,50,53], and one study utilized positive youth development theory [51]. Other analytical frameworks employed included program effectiveness [52], critical pedagogy and collaborative action research [54], adult learning theories [59], and socialization theories [57,58].

### Topic summary

**Fig 2:**
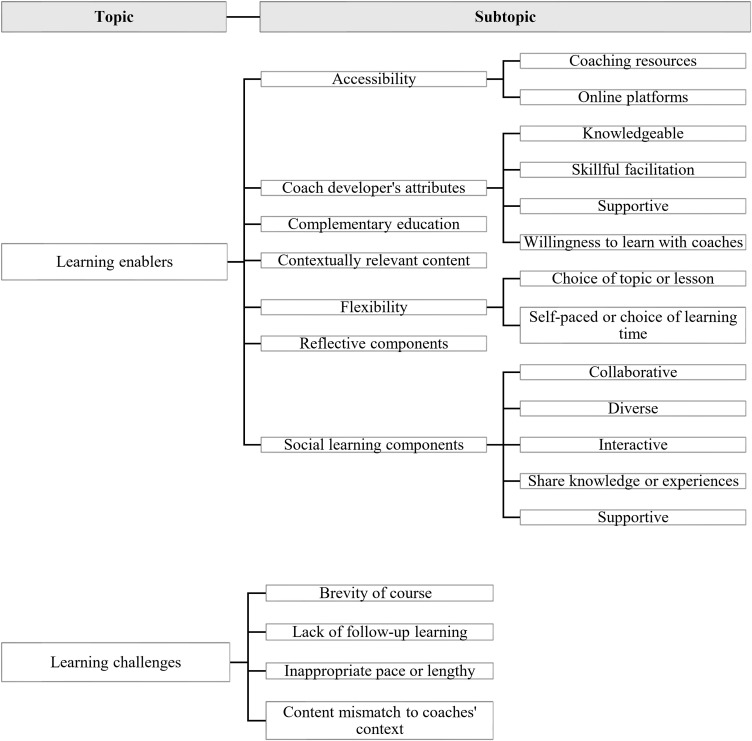
Organisational map of the topics and subtopics (Learning enablers and Learning challenges) based on the qualitative analysis of the included studies.

**Fig 3:**
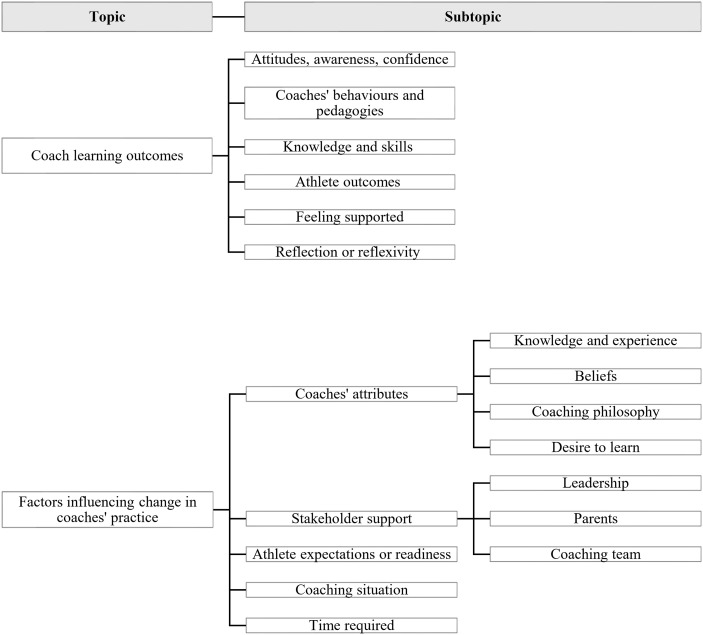
Organisational map of the topics and subtopics (Coach learning outcomes and Factors influencing change in coaches’ practice) based on the qualitative analysis of the included studies.

Figs 2 and 3 present an overview of the qualitative topics and their respective subtopics identified through the analysis of the eleven included studies. In summary, the analysis revealed four topics: (a) learning enablers; (b) learning challenges; (c) outcomes; (d) factors influencing change in coaches’ practice. These summary topics will be explicated in turn.

### Learning enablers

Accessibility (three studies, 27.3%) emerged as a key enabler of learning, characterised by increased access to either quality coaching resources or online learning platforms [51,54,56]. Remote learning was noted to enhance “time efficiency” [56 p. 74] in learning facilitating access to coaching resources at no cost. Furthermore, some coaches revisited these resources to reinforce their understanding of coaching topics [54] and integrate them into their coaching practice [51,56]. For example, Strachan et al. [51] found that learning was facilitated when coaches utilised the Project SCORE! Website and supplemented this with their own resources to implement the various themes and lessons in their coaching.

Five studies (45.5%) emphasised the positive impact of an effective coach developer on learning. For instance, coach developers were skilful in promoting dialogue between other coaches with differing viewpoints, stimulating thinking on alternative ideas, providing coaching feedback, raising coaches’ awareness, and “sense making” [54 p. 73] on topics relevant to youth participation coaching (e.g., holistic youth coaching). For example, during a reflective conversation with her coach developer, one coach was prompted to challenge her existing assumptions about coaching:

*“Thinking back, I just feel coaching stuﬀ that I had seen others do and kind of thought why do I need to do anything diﬀerent. And I don’t know if I do, and I am not saying what I have been doing is wrong but I ﬁnd myself asking more about why I am doing these things now.”* [54]

Coaches also highlighted several desirable attributes including knowledge of both the sport coached and pedagogical principles [58], as well as a willingness to support and learn alongside them [54,59]. The following quotes illustrate the value of coach developers when they function as co-learners and supportive partners in the learning process:

*“I felt supported because [facilitator] was very involved and passionate about facilitating our communities of practice discussions and maintaining open lines of communication”* [59]

*“Taking an interest in what I am doing and oﬀering advice, I think. It’s being invested in. I talk with other coaches but not in a way where I feel I am being helped with my coaching”* [54]

Coaches emphasised the importance of ‘continuing and complementary education’ [55] to support the development of their coaching knowledge. This aligns with findings in seven studies (63.6%) where coaches highlighted the importance of contextually relevant content. Depending on their existing knowledge, certain topics were perceived as ‘new’ (e.g., coaching pedagogy [57,58]) or ‘familiar’ (e.g., autonomy-supportive coaching [50,53]). Furthermore, coaches valued content that was ‘easy to apply’ [52] often with ‘minimal preparation required’ [51] for integration into their practice.

Flexibility in learning was also identified as a key learning enabler by coaches (four studies, 36.4%). This encompassed having choice in learning topics and the ability to learn at their own pace. For example, one coach said, “I really enjoyed the freedom to go and search and learn about things that I wanted to learn about without being told I had to learn certain things” [54]. This flexibility was beneficial for coaches engaging with CEP featuring self-paced components [51,56] and when offered varied ‘call time options’ for online discussions [59].

Coaches valued learning approaches that incorporated reflection (five studies, 45.5%) and social learning activities (seven studies, 63.6%). Reflection was facilitated through various methods, including peer discussion (e.g., [53]) and reflective conversations with mentors [54]. Coaches reported that learning alongside fellow coaches enhanced their understanding, knowledge, or awareness of discussed topics. This may be fostered through collaborative work (e.g., [52]) or sharing at conferences and forums [55]. These discussions provided opportunities to gain perspectives from coaches with similar experiences [56] as well as diverse perspectives from coaches in different sports [59]. Furthermore, coaches noted knowledge sharing was promoted in an environment that is supportive [57,59] and interactive [53].

### Learning challenges

A significant learning challenge identified by coaches was the brevity of courses (five studies, 45.5%). Specifically, within youth grassroots football, over half of the coaches surveyed advocated for extending the under 12 and under 10 certification courses, citing insufficient preparation for their coaching roles [55]. This concern was echoed by other coaches who noted a “lack of time invested in how to actually teach” [58 p. 18], and the need for follow-up sessions to consolidate learning as the initial lectures “tend to slip” [53 p. 126].

Furthermore, the lack of follow-up after attending CEP was a recurring concern, particularly in shorter courses (less than a day) [52,53,57,58]. Some coaches, suggested that follow-up could involve “more course sessions to allow for a more in-depth exploration of the topics” [52] or practical learning sessions at their respective clubs:

*At the workshop, we had some thoughts regarding what we wanted to carry out in a training session… but it’s difficult, not difficult, rather a barrier to doing this, when you are all alone. So I think that, if the club had organized this, not to meet every week, but maybe once a month, for instance, discussing topics related to the modules released, meet and talk, to exert this mild pressure to carry out the plans…* [52]

*“The club could have facilitated a meeting once or twice a month for sharing experiences and keeping focus”* [53]

Conversely, coaches also identified length or pacing issues as a learning challenge [52,59]. To address this, Swartz et al. [59] reduced the CEP timeline from 26 weeks to 14 weeks in the second phase of their action research, aiming to mitigate coach fatigue. A further challenge experienced by coaches was the mismatch between learning content and their specific coaching context [51,55]. For example, one coach suggested that the holistic youth coaching lesson package contained “abstract concepts” (p. 113) that needed to be adapted for younger age groups [51], implying a need for more practical and age-appropriate examples.

### Coach learning outcomes

Following participation in coach education and learning activities, coaches reported a range of positive outcomes. Notably, improved attitudes, awareness and confidence were frequently mentioned (eight studies, 72.7%) alongside an influence on coaches’ behaviours and adoption of new pedagogies (eight studies, 72.7%). As one youth grassroots football coach expressed, this education prompted a shift in their perspective on coaching:

*“First, what I remember (from the workshop), is to give freedom to the players, rather than always telling them what to do, especially the freedom of expressing themselves, letting them talk, and ask questions. I used to do this before as well, but it is important to get reminded in order to motivate each child to sustain their motivation for football”* [50]

Another outcome reported by coaches was that the CEP influenced their coaching behaviour(s) and pedagogical approach(es). Most of these studies reported positive changes in coaches’ practices that were largely in line with the CEP’s aims (e.g., autonomy-supportive strategies, athlete-centred coaching). The following quotes illustrate this:

*“I have used many of the strategies in coaching Gen Z to include more opportunities for them to make decisions.”* [59]

*“After a game, I ask what they think went well, what went poorly and also if they have any idea of how we can work on this… and then they come with some suggestions”* [50]

Moreover, to further examine the impact of CEP on coaching behaviours, four studies [49,54,57,58] employed mixed-method, longitudinal designs (e.g., pre- and post-CEP) to validate qualitative (e.g., interviews) with quantitative (e.g., systematic observations) findings. These studies yielded mixed results. For example, Barnes and Curtner-Smith [58] found that only one of the two coaches in their case study demonstrated significant changes in their pedagogical approach (i.e., shift from direct to indirect teaching styles). Similarly, changes in coaching behaviours (e.g., questioning, feedback) and pedagogies varied among the three coaches investigated in Cope et al.’s [54] study.

Coaches from five studies (45.5%) reported gaining knowledge and skills during their CEP experiences, including content knowledge, managerial and planning skills [58], communication skills [49,50,56], and an understanding of how to influence athlete motivation [53]. Furthermore, coaches in three studies (27.3%) observed positive impacts on athletes’ outcomes, such as increased enjoyment [51] and greater interest in practice’ sessions [49]. The subtopic of feeling supported was highlighted by coaches in three studies (27.3%). This support stemmed from having a supportive network of fellow coaches [57] and they desired “feeling connected to a larger community of coaches” [59 p. 14]. Finally (i.e., *reflection or reflexivity* subtopic), one study explicitly noted that CEPs helped coaches develop their ability to critically reflect on their coaching practice [54].

### Factors influencing change in coaches’ practice

Participant coaches across the included studies identified several factors influencing changes in their coaching practice following coach learning experiences. Analysis revealed a range of coaches’ attributes (i.e., *knowledge and experience, beliefs, coaching philosophy, desire to learn*) that influenced the extent to which they applied new coaching principles and ideas learned at coach education and learning experiences. The findings from five studies (45.5%) indicate that coaches’ existing coaching knowledge and experiences influenced how readily they adopted coaching principles learned through both formal or informal learning opportunities. For example, Barrero et al. [55] found significant differences in the application of coaching principles among youth grassroots football coaches based on their prior sport coaching education and perceived readiness to coach, as revealed by a survey of 153 coaches. Prior experience was associated with the adoption of autonomy-supportive coaching behaviours [49] and the integration of new lesson ideas with existing knowledge (and resources) [51].

The subtopic of coach beliefs encompasses their perspectives on specific coaching approaches (e.g., indirect teaching styles, holistic youth coaching strategies) and their perception of the validity of coaching knowledge. For example, Barnes and Curtner-Smith [57,58] noted that a coach’s eventual application of learned pedagogy may be contingent by their individual beliefs (e.g., prioritising athlete skill development). Furthermore, beliefs were identified as key in determining whether newly learned coaching ideas are accepted without critical evaluation, particularly among less experienced coaches [54]. This is exemplified in the following interview excerpt:

*“There were a few coaches who had commented on a discussion I posted last week. I had a look on their proﬁles and they seemed experienced and knew what they were talking about so I took on board what they said and used some of the ideas this week.”* [54]

Four studies (36.4%) emphasised the importance of a coach’s philosophy in determining the effectiveness of interventions like the CEP. Larsen et al. [50] found that a shared understanding of the CEP principles was crucial for coaches to consistently implement the intended coaching strategies shared in the course. Swartz et al.’s [59] study also highlights the role of perceived organisational support, attributing coaches’ desire to learn to a perceived lack of support. The subtopic of *athlete expectations or readiness* was evident in the findings from five studies (45.5%). For example, coaches varied their coaching approach (e.g., direct teaching styles) based on their athletes’ prior experience [58] or perceived skill level [57]. Similarly, Langdon et al.’s [49] research revealed that coaches faced challenges in implementing autonomy-supportive strategies with youth athletes, which included efforts to maintain engagement, “waiting for the athletes to answer questions” (p. 9), and “avoid controlling language” (p. 9) even when faced with disobedience.

Stakeholder support was a key factor influencing coaches’ implementation of learned coaching ideas and strategies. This encompasses several subtopics: *leadership* (four studies, 36.4%), *parents* (four studies, 36.4%) and *coaching team* (two studies, 18.2%). Søvik et al.’s [52] study indicated that a “lack of encouragement from club leadership” (p. 168) and challenges in achieving shared understanding with their co-coaches hindered the application of CEP coaching principles. Furthermore, coaches reported that parents’ expectations, such preparing child-athlete for high school try-outs [57] or focusing on results [52], significantly influenced their decision to implement learned coaching principles. Situational factors also influenced the application of coaching principles (four studies, 36.4%). Coaches adjusted their coaching approach (e.g., autonomy-supportive strategies, direct or indirect teaching styles) based on whether they were in league games or non-game settings [49] or ‘practice or games’ situation [57]. Finally, the *time required* for effective implementation was noted in three studies (27.3%). This included the time required in strategy planning [52] or time to implement holistic youth coaching sessions [51].

## Discussion

Based on the inclusion and exclusion criteria, eleven articles were identified and analysed for this review. While a significant body of research exists in elite sports coach learning (e.g., [10]) and development (e.g., [23]), athlete development (e.g., [62]), and youth sports participation for adolescents (e.g., [63]), the literature examining the education and learning of youth participation coaches appears to be lacking. Youth participation coaches play a crucial role in introducing athletes to sport, facilitating early psychomotor skill development [6], athlete retention, and overall athlete development [8]. Concomitantly, a coach’s capacity for learning and applying relevant knowledge is fundamental to their effectiveness. Therefore, examining the literature on youth participation coach learning would provide valuable insights into aspects such as coaching practice within youth sport programmes (e.g., [64]), ongoing learning (e.g., [34]) and retention (e.g., [33]) among these coaches. The findings from these eleven studies are discussed in the following paragraphs.

### Study characteristics and participant profile

The scoping review has summarised and identified some characteristics of the youth participation coach education and learning literature. Based on geographical location, there is an underrepresentation of studies examining coaches outside of western countries and individual sports. This finding aligns with Lyle and Crespo’s [65] notion that “knowledge diffusion via the academic community is largely dominated by Euro-North American academics” (p. 369). Further, the results may also partly be attributed to the inclusion criteria of the review (i.e., articles in English), which suggests that non-English speaking coaches may have been excluded or underrepresented in the research. In terms of sport type investigated, there is little known in the literature that explains the lack of research of youth participation coach education and learning of coaches practicing in individual sports. The prevalence of football-focused studies (six) aligns with findings from coach education program reviews, which identify football as the most researched sport [66]. This likely reflects football's widespread popularity globally – in terms of viewership, participation, and support – potentially influencing resource allocation towards its development, including coach education [67].

Results also demonstrate a moderately larger representation of coaches who are volunteers (six studies) with some coaching experience (3–10 years on average). Additionally, the review highlighted several studies that did not indicate coaches’ status (four studies) and clearly differentiate coaches’ prior coaching qualifications (eight studies). While volunteer coaching is common in youth participation sport in some countries (e.g., Australia [33]; United Kingdom [68]) and regions (e.g., European Union [32]), it should not be taken for granted that the learning needs or motivations of coaches of other statuses (i.e., part-time, full-time) are similar. Considering the influence of coaches’ background (e.g., coaching status, experience, education) on their learning and practice [25,69,70], there appears to be limited understanding of how coaching knowledge and experience may shape youth participation coaches’ learning experiences. Moreover, scholars argue that the view of coach learning should extend beyond previous assumptions of just knowledge transfer and through interaction with various sources [12,13], learning situations and contexts [16,27]. Indeed, in the current era of globalisation and the drive to actualise how coaching knowledge can be effectively created and disseminated globally, it is crucial that research must consider “significant cultural differences” [65 p. 357] across countries, as well as sporting systems to advance our understanding of coach learning. This lack of clarity hinders our ability to determine how these coaches learn and subsequently, how best to support their preparation for their unique roles [26,28,29]. Indeed, Nelson et al.’s [71] study of community coaches with varied employment statuses (i.e., full-time, part-time, volunteer) found that part-time or volunteer coaches used specific management techniques to cope with fatigue from their primary employment.

Based on the methodology of the reviewed studies, most (nine) studies explicitly stated the researchers’ philosophical assumptions and theoretical frameworks used to interpret findings. This aligns with recommendations for more representative research designs that incorporate theoretical perspectives (e.g., social learning theory, adult learning theory) which can offer more nuanced explanations of how youth participation coaches learn [2,72]. Based on research method, qualitative approaches were predominantly adopted, which corroborates with findings from a systematic review of coach education programs [73]. While there were deep insights gathered (i.e., mixed or qualitative approaches) and some research that were better aimed at contributing to practice (e.g., action research [59]), it is also apparent that research gaps exist. Specifically, the lack of breadth of findings due to small sample sizes, the lack of quantitative focus, as well as the absence of other possible research paradigms to explain coach learning (e.g., [74], limits our understanding in this area of study.

### Design of youth participation coach education programs

Analysis informed by CEP design, highlights a need for future research focused on how informal learning sources influence the knowledge and practice of youth participation coaches. While formal learning has received considerable attention in coach learning research [21,38,75], less is understood about the role of informal learning. Furthermore, research indicates that CEPs typically contribute a relatively small portion to coaches’ acquisition of knowledge and skills [2,14,15]. In retrospect, the debate over the superiority of formal versus informal coach education is largely unproductive [11]. Therefore, future research should focus on understanding coaches’ preferences for ‘ongoing and complementary education’ [55] and how both formal and informal learning contribute to their overall development.

While literature suggests that customized learning within smaller CEPs has the potential to positively impact coaches [38,76], the effectiveness of these programs remains largely unexplored. Conversely, research on large-scale CEPs has often focused on limitations in delivery and evidence-based content, leading to concerns about their impact on coaching practice [21,77]. Despite these criticisms, large-scale CEPs remain an important part of coaches’ professional development as they offer coach accreditation upon successful completion [78]. From a practical perspective, this accreditation provides sport organisations or clubs with assurance of coaches’ competency [78]. However, research examining the influence of large-scale CEP participation on individuals remains limited; only two studies have examined this specific aspect (two coaches and one coach respectively) [57,58]. Hence, future research should prioritise understanding the experiences and perspectives of coaches participating in large-scale CEPs to gain a more comprehensive understanding of their potential to support coaching practice [30,77].

The findings from this review determined that coaches commonly desired CEP content to be practically and contextually relevant to their practice. Based on the analysis, *contextually relevant content* (seven studies) was deemed as a learning enabler. In contrast, *content mismatch to coaches’ context* (two studies) was considered a learning challenge. These findings illustrate the argument that the learning content delivered at CEPs, should match the specific coaching context [25,79] as well as being easily applicable to their practice. Hence, to better prepare youth participation coaches for their unique coaching roles [26], literature suggests CEPs should provide relevant content (e.g., creating a motivational climate for young athletes [80], holistic coaching principles [63], and game-based coaching approaches [81]) as well as develop specific competencies (e.g., session planning, organisation and management, and working with parents and/or sport administrators [3]). Considering the learning needs of youth participation coaches can be highly varied based on their specific coaching context, it does appear that the literature is unable to provide sufficient explanation of how coaches’ needs may be met [37,82].

The attributes of effective coach developers tend to align with established principles in coach education that span across diverse coaching contexts [21,75,83]. Practically, as this review has highlighted, this has an ongoing positive effect on learning due to the significant influence coach developers have on their coaches’ development. For example, they affect active learning environments that encourage maximum participation and engagement [84], and they encourage developing coaches to critically inquire into coaching challenges [85] such as working with athletes of diverse playing abilities or coaching under resource constraints (e.g., limited equipment or facilities). Overall, substantial evidence indicates that the qualities of effective coach developers are fundamental to impactful learning outcomes.

All CEPs included within this research utilised online platforms. Based on the perspectives of coaches, they welcome the flexibility to learn at their own pace and additional time to dive deeper into the content covered beyond the duration of the CEP [56]. Moreover, remote access to learning resources provided coaches with readily available reference materials to support their practice (Strachan et al., 2016). This finding aligns with coach learning research, that advocates online platforms as a medium to promote social learning [14], self-paced learning [79], and sharing of online resources [15]. While there is substantial evidence of coaches’ receptivity towards online and hybrid modes of learning, there is little evidence of the use of specific digital tools (e.g., online discussion boards) combined with learning strategies (e.g., reflection, providing feedback) that effectively contributes to coaches’ learning [19,38].

The duration of CEPs significantly influences coaches’ learning. Coaches in shorter programs (i.e., less than a week) often want more time to deepen their understanding of coaching principles [49,52]. Additionally, they emphasised the need for post-CEP reflection activities to reinforce learned information [57,58]. In contrast, longer programs have been described as too drawn out [59] and the learning pace could be improved through more timely follow-up activities [52]. Research consistently highlights that learning requires sufficient time and dedicated space for coaches to process content delivered at CEPs [16,77]. Indeed, a study on learner-centred strategies in a grassroots football coaching course identified that limited learning time restricted opportunities for coaches to explore their own coaching practice and learning needs [86]. Hence, understanding how to optimise learning time for youth participation sports coaches is essential for their ongoing development.

### Effectively supporting youth participation coaches in learning

The review highlights the recurring theme of ‘support’ needed for youth participation coaches to learn and thrive in their practice. From the analysis of the eleven studies, it appears there are a range of factors that may support (or thwart) youth participation coaches in their learning and to apply their knowledge in practice. In their learning process, there is some indication that coaches’ desire for support may be facilitated by coach developers [54] and their community of coaches [57]. Evidence suggests that collaborative, group-based learning activities can enhance coaches’ confidence when these activities are well-facilitated [52]. From a social learning perspective, the value of peer relationships in this process is evident: coaches benefit not only from sharing similar experiences and challenges [56] but also from exposure to diverse perspectives from coaches in other sports [59]. Additionally, a non-competitive and supportive environment appears vital for engagement, with coaches highlighting the importance of enjoyable, collegial interactions [52,59]. These findings align with the broader social learning theory [87,88], which emphasises the role of collaboration and shared experience in professional development.

Youth participation coaches frequently report feeling unsupported in their professional development, particularly when there is no follow-up after participating in a CEP [53] or when long-term learning pathways are absent [36]. Their ability to apply new and existing knowledge is influenced by multiple stakeholders, including club leadership, parents, co-coaches, and athletes [52,58]. Moreover, coaches must navigate complex dynamics with stakeholders, entrenched cultural norms [53], specific coaching scenarios [49], and their own personal beliefs [58]. These factors collectively shape the extent to which learned coaching principles are implemented in practice. Alongside these factors, the literature indicates that sports clubs and organizations running youth sport programs often operate on tight budgets. This limits opportunities for developing their youth coaches [63] and makes it challenging to meet the diverse needs of a coaching workforce given resource constraints [22]. While scholars advocate for domain-specific approaches to coach education [1,7], there is a notable lack of research exploring the day-to-day realities of youth participation coaching. This gap highlights the need for empirical studies that examine how these coaches navigate their unique challenges, including limited resources, diverse work environments, and competing stakeholder expectations.

### Evidencing the impact of coaches’ learning and practice

The scoping review revealed that measuring impact on youth participation coach learning and practice is essential to develop a better understanding of how coaches may be supported through coach education. In developing this understanding, the studies adopted a mix of research approaches to explore, explain or describe changes in coaches’ behaviours or practices resulting from their learning experiences. Additionally, coaches reported a range of outcomes (e.g., athlete outcomes, knowledge and skills) while they shared a range of factors (e.g., athlete expectations and readiness, coaches’ attributes) that would influence their implementation of learned coaching strategies and ideas. While three studies [49–51] reported improvements in athlete behaviours — such as increased enjoyment [51] — they offered limited insight into how coaches applied their learning to achieve these results. Furthermore, literature identifies a range of desirable athlete outcomes (e.g., competence, confidence, connection, character), that coaching can foster [7]. Given that a coach’s primary role, regardless of context, is to facilitate athlete learning, further research into this aspect would significantly contribute to the youth participation coach education and learning literature.

Scholars in all the reviewed studies agree that CEP should aim at shifting coaches’ existing assumptions about coaching. To effectively achieve this, research suggests designing CEPs with coaches at the centre of learning [16,83]. Additionally, it should be acknowledged that coaches have pre-existing orientations to learning shaped by their past experiences and contexts [82,89]. Coaches approach any learning experience with a preconceived set of values and beliefs formed through their socialisation. This means they actively decide whether to ‘accept’ or ‘reject’ new coaching knowledge presented during CEPs [25]. As Barnes and Curtner-Smith’s [57] study demonstrates, even with similar coach education, coaches can have different outcomes based on their prior socialization. Similarly, Cope et al. [54] found that novice coaches may uncritically accept coaching principles from more experienced coaches. Therefore, future research should focus on gaining deeper insights into how coaches’ dispositions influence learning.

In summary, effective coach learning is a complex phenomenon influenced by a range of contextual factors (e.g., stakeholder support, coaches’ role). The literature has emphasised considering subtle differences between countries, sports, coaches’ background (knowledge, experience, accreditation) and the lack of representative approaches in coach learning research. These findings suggest several practical implications for stakeholders involved in coach development. Specifically, sport organisations and governing bodies should allocate financial resources and time to enable coaches to pursue professional development opportunities independently [22]. CEPs should move away from didactic, ‘top-down’ content delivery and instead foster critical reflection on personal practice [82,85]. Furthermore, coach education curricula should be designed to reflect the diverse contexts in which coaches operate [37]. Despite these suggestions, the limited evidence available in youth participation coach education and learning literature hinders our understanding of how these coaches learn and can be best supported. Given the limited number of studies, research designs and coaching contexts investigated, the findings of this review offer only a preview of these factors. Consequently, more research is required to examine learning in relation to the coaches’ role within specific contexts.

### Limitations

While this scoping review provides valuable insights into youth participation coach education and learning, several limitations should be acknowledged. Firstly, the scope was limited to peer-reviewed articles published in English, after 2010, meaning relevant non-English or earlier studies were excluded [42]. Secondly, despite employing a variety of search terms such as ‘volunteer’, ‘grassroots’, ‘participation’, ‘recreation’, the exclusion of ‘community’ may have inadvertently narrowed the scope. This decision aimed to avoid an overwhelming number of results beyond the field of sport coaching. However, it does highlight a potential challenge in capturing the full range of contexts where participation coaches operate. Consistent with scoping review methodology, this study focused on presenting insights according to research activity, major findings, and research gaps [39] rather than assessing the quality or methodological robustness of the included studies. The current scoping review does not include a systematic appraisal of the methodological rigour of the included studies. While this approach allows for a broad overview of the literature, it inherently limits our ability to critically evaluate the strengths and weaknesses of individual studies and synthesise findings with a high degree of certainty. Consequently, the conclusions drawn from this review should be interpreted with caution regarding robustness and generalisability. Finally, the review acknowledges that a wider range of research paradigms beyond those operating within Western contexts and team sports may offer alternative perspectives on coach learning, further limiting the generalisability of our findings to non-western environments or individual sports [74].

### Implications for future research

To further advance our understanding of youth participation coach education and learning, future research should address several key gaps. Firstly, studies should prioritise inclusivity by involving coach participants from under-represented contexts such as non-western countries. Research in these contexts will significantly advance coach learning by acknowledging the unique sporting system and coach education structures influenced by the prevailing culture within the country [65]. Secondly, addressing the methodological limitations of existing studies is crucial. Future studies should utilise larger, more representative samples to enhance the generalisability of findings. Specifically, comparative analysis across coaches in different countries, sports and with varying backgrounds (knowledge, experience, accreditation) would contribute to broader insights into the field. Finally, future investigations should explore how coach learning translates to specific athletes’ outcomes (e.g., competence, confidence, connection, character [7]). Providing such insight will enhance our understanding of how youth participation coaches play an educative role in developing their athletes holistically using developmentally appropriate coaching strategies.

The findings from the review also prompt researchers to consider studies that examine specific CEP design features that support youth participation coaches’ practice. Given the transforming digital landscape, this includes investigating the effectiveness of various learning modes (online, hybrid) and specific digital tools (e.g., online discussion boards) on coach learning. Another important area for research is determining the optimal duration of CEPs to facilitate learning, particularly addressing the time constraints often faced by youth participation coaches [33]. Furthermore, deeper investigations are needed into the requisite knowledge and skills for coaches in their roles. The review highlighted a limited understanding of key competencies such as autonomy-supportive strategies and holistic youth coaching practices [26]. Therefore, exploring alternative CEP formats [54] that provide insights into how coaches learn would be a valuable contribution to the field. This research will inform the development of comprehensive, contextually relevant CEP that support coaches’ overall development. It will also assist coach development bodies in optimising specific CEP offerings to ensure cost-effectiveness without compromising quality.

## Conclusion

This scoping review contributes to the growing body of research in sport coaching by synthesising insights into youth participation coach education and learning. The findings from the eleven included studies highlight key areas of the investigation, including the influence of CEP on coach development, while also identifying critical gaps in the literature. Specifically, further research is needed into the experiences of coaches from non-western contexts, individual sports, and diverse coaching backgrounds. Findings also underscore the importance of designing CEP’s with contextually relevant content, flexible delivery methods, and accessible learning opportunities to better meet the needs of youth participation sports coaches. However, the review reveals that coaches face significant challenges in applying learned coaching principles into their practice. This is often due to limited stakeholder support, resource constraints and conflicting cultural expectations. Ongoing organisational and peer support from key stakeholders (e.g., coach education providers, club leadership, co-coaches) are key factors identified as essential for coaches’ growth and effectiveness.

Moving ahead, future research should focus on exploring culturally diverse coaching environments, employing larger and more representative coach samples, and investigating the design and delivery of CEP’s tailored to the specific needs of the youth participation sports coach. Additionally, there is a pressing need to examine how youth participation coach education and learning translate into tangible athlete outcomes such as competence, confidence, connection and character [7]. By addressing these gaps, future studies can provide actionable insights to advance the field of youth participation coach education and foster the development of more effective coaching practices.

## Supporting information

S1 File Preferred Reporting Items for Systematic reviews and Meta-Analyses checklist. (DOCX)

S1 Table Study context and participant characteristics. (DOCX)

S2 Table Coach Education Program (CEP) characteristics. (DOCX)

## References

[1] Lyle J, Cushion CJ. Sport coaching concepts: A framework for coaching practice. London, England: Routledge; 2017.

[2] Trudel P, Gilbert W. Coaching and coach education. Handbook of physical education. Sage; 2006. p. 516–39.

[3] Cronin C, Armour KM. Lived experience and community sport coaching: A phenomenological investigation. Sport Educ Soc. 2015;20(8):959–75. doi: 10.1080/13573322.2013.858625

[4] Mallett CJ, Rynne S. Changing Role of Coaches across Development. Routledge Handbook of Sport Expertise. Routledge; 2015. p. 394–403.

[5] Côté J, Abernethy B. A Developmental Approach to Sport Expertise. The Oxford Handbook of Sport and Performance Psychology. Oxford University Press; 2012. p. 435–47.

[6] Cope E, Plimmer N. Coaching children: A guide to maximising their enjoyment. Sports Coaching. Routledge; 2019. p. 157–67.

[7] Côté J, Gilbert W. An Integrative Definition of Coaching Effectiveness and Expertise. Int J Sports Sci Coach. 2009;4(3):307–23. doi: 10.1260/174795409789623892

[8] Côté J, Turnnidge J, Vierimaa M. A personal assets approach to youth sport. Routledge handbook of youth sport. Routledge; 2016. p. 243–55.

[9] Gilbert W, Côté J. Defining coaching effectiveness: a focus on coaches’ knowledge. Routledge handbook of sports coaching. Routledge; 2013. p. 147–59.

[10] Walker LF, Thomas R, Driska AP. Informal and nonformal learning for sport coaches: A systematic review. Int J Sports Sci Coach. 2018;13(5):694–707. doi: 10.1177/1747954118791522

[11] Mallett CJ, Trudel P, Lyle J, Rynne SB. Formal vs. Informal Coach Education. Int J Sports Sci Coach. 2009;4(3):325–64. doi: 10.1260/174795409789623883

[12] Erickson K, Bruner MW, MacDonald DJ, Côté J. Gaining Insight into Actual and Preferred Sources of Coaching Knowledge. Int J Sports Sci Coach. 2008;3(4):527–38. doi: 10.1260/174795408787186468

[13] Lemyre F, Trudel P, Durand-Bush N. How youth-sport coaches learn to coach. Sport Psychol. 2007;21(2):191–209.

[14] Stoszkowski J, Collins D. Sources, topics and use of knowledge by coaches. J Sports Sci. 2016;34(9):794–802. doi: 10.1080/02640414.2015.1072279 26222481

[15] Van Woezik RA, McLaren CD, Côté J, Erickson K, Law B, Lafrance Horning D, et al. Real Versus Ideal: Understanding How Coaches Gain Knowledge. Int Sport Coach J. 2022;9(2):189–202. doi: 10.1123/iscj.2021-0010

[16] Trudel P, Culver D, Werthner P. Looking at coach development from the coach-learner’s perspective: Considerations for coach development administrators. Routledge handbook of sports coaching. Routledge; 2013. p. 375–87.

[17] Callary B, Brady A, Kiosoglous C, Clewer P, Resende R, Mehrtens T, et al. Making Sense of Coach Development Worldwide During the COVID-19 Pandemic. Int J Sport Commun. 2020;13(3):575–85. doi: 10.1123/ijsc.2020-0221

[18] Santos F, Cardoso A, Pereira P, Strachan L. Coach Training Within the Covid-19 Pandemic: Challenges and Potential Pathways. Front Psychol. 2021;12:570706. doi: 10.3389/fpsyg.2021.570706 33995165 PMC8116699

[19] Cushion CJ, Townsend RC. Technology-enhanced learning in coaching: a review of literature. Educ Rev. 2019;71(5):631–49. doi: 10.1080/00131911.2018.1457010

[20] Jones T, Allen J, Macdonald S. The “Face” of Coach Development: A Systematic Review of the Role of the Coach Developer. Int Sport Coach J. 2024;11(1):1–19. doi: 10.1123/iscj.2022-0017

[21] Wang Z, Casey A, Cope E. Coach experiences of formal coach education developed by national governing bodies: a systematic review. Phys Educ Sport Pedagogy. 2025;30(3):351–63. doi: 10.1080/17408989.2023.2230235

[22] Nash C, Sproule J, Horton P. Continuing professional development for sports coaches: a road less travelled. Sport Soc. 2017;20(12):1902–16. doi: 10.1080/17430437.2017.1232414

[23] Trudel P, Gilbert W, Rodrigues F. The journey from competent to innovator: Using appreciative inquiry to enhance high performance coaching. Int J Appreciat Inq. 2016;18(2).

[24] Gilbert WD, Trudel P. Learning to Coach through Experience: Reflection in Model Youth Sport Coaches. J Teach Phys Educ. 2001;21(1):16–34. doi: 10.1123/jtpe.21.1.16

[25] Stodter A, Cushion CJ. What works in coach learning, how, and for whom? A grounded process of soccer coaches’ professional learning. Qual Res Sport Exerc Health. 2017;9(3):321–38. doi: 10.1080/2159676x.2017.1283358

[26] Penney D, Jeanes R, O’Hara E, Magee J. Community Sport Coaches as Policy Actors. Community Sport Coaching. Routledge; 2022. p. 45–60. doi: 10.4324/9781003159063-3

[27] Cushion CJ, Nelson L. Coach education and learning: Developing the field. Routledge handbook of sports coaching. Routledge; 2013. p. 359–74.

[28] Jones GJ, Edwards MB, Bocarro JN, Svensson PG, Misener K. A community capacity building approach to sport-based youth development. Sport Manage Rev. 2020;23(4):563–75. doi: 10.1016/j.smr.2019.09.001

[29] Crisp P. Leadership, bridging, and group-game engineering: guidelines for community sport coaches. Int Sport Coach J. 2020;7(2):229–38.

[30] Nash C, Sproule J. Coaches perceptions of their coach education experiences. Int J Sport Psychol. 2012;43(1):33.

[31] Battaglia A, Kerr G, Buono S. Coaches’ perspectives of the influence of safe sport-related education. Sport J. 2024;1.

[32] Lara-Bercial S, Calvo G, North J, Moustakas L, Petry K. The EU Coaching Landscape Report 2020. 2020.

[33] Baxter H, Misener KE. Retaining volunteer coaches in child and youth sport. Routledge Handbook of Coaching Children in Sport. Routledge; 2022. p. 412–20.

[34] Vargas-Tonsing TM. Coaches’ Preferences for Continuing Coaching Education. Int J Sports Sci Coach. 2007;2(1):25–35. doi: 10.1260/174795407780367186

[35] Wiersma LD, Sherman CP. Volunteer youth sport coaches’ perspectives of coaching education/certification and parental codes of conduct. Res Quart Exerc Sport. 2005;76(3):324–38. 10.1080/02701367.2005.10599303 16270709

[36] O’Connor D, Bennett B, Perry K, Fyall G, Rogers A, Morley D. Coach development in Australia and Aotearoa New Zealand: Evolutions and current directions. The Routledge Handbook of Coach Development in Sport. Routledge; 2024. p. 22–44.

[37] Holdom T, Nichol A, Ives B. Recognising, addressing and supporting the challenging nature of community sport coaching work: potential ways forward for research and practice. Sports Coach Rev. 2024;13(2):265–76. doi: 10.1080/21640629.2024.2335432

[38] Li L, Olson HO, Tereschenko I, Wang A, McCleery J. Impact of coach education on coaching effectiveness in youth sport: A systematic review and meta-analysis. Int J Sports Sci Coach. 2024;20(1):340–56. doi: 10.1177/17479541241283442

[39] Arksey H, O’Malley L. Scoping studies: towards a methodological framework. Int J Soc Res Methodol. 2005;8(1):19–32. doi: 10.1080/1364557032000119616

[40] Munn Z, Peters MDJ, Stern C, Tufanaru C, McArthur A, Aromataris E. Systematic review or scoping review? Guidance for authors when choosing between a systematic or scoping review approach. BMC Med Res Methodol. 2018;18(1):143. doi: 10.1186/s12874-018-0611-x 30453902 PMC6245623

[41] Brien SE, Lorenzetti DL, Lewis S, Kennedy J, Ghali WA. Overview of a formal scoping review on health system report cards. Implement Sci. 2010;5:2. doi: 10.1186/1748-5908-5-2 20205791 PMC2823624

[42] Grant MJ, Booth A. A typology of reviews: an analysis of 14 review types and associated methodologies. Health Info Libr J. 2009;26(2):91–108. doi: 10.1111/j.1471-1842.2009.00848.x 19490148

[43] Rumrill PD, Fitzgerald SM, Merchant WR. Using scoping literature reviews as a means of understanding and interpreting existing literature. Work. 2010;35(3):399–404. doi: 10.3233/WOR-2010-0998 20364059

[44] Levac D, Colquhoun H, O’Brien KK. Scoping studies: advancing the methodology. Implement Sci. 2010;5:69. doi: 10.1186/1748-5908-5-69 20854677 PMC2954944

[45] Tricco AC, Lillie E, Zarin W, O’Brien KK, Colquhoun H, Levac D, et al. PRISMA Extension for Scoping Reviews (PRISMA-ScR): Checklist and Explanation. Ann Intern Med. 2018;169(7):467–73. doi: 10.7326/M18-0850 30178033

[46] Peters MDJ, Godfrey CM, Khalil H, McInerney P, Parker D, Soares CB. Guidance for conducting systematic scoping reviews. Int J Evid Based Healthc. 2015;13(3):141–6. doi: 10.1097/XEB.0000000000000050 26134548

[47] Covidence. Covidence systematic review software. [Computer software]. In press. 2024.

[48] Harrison H, Griffin SJ, Kuhn I, Usher-Smith JA. Software tools to support title and abstract screening for systematic reviews in healthcare: an evaluation. BMC Med Res Methodol. 2020;20(1):7. doi: 10.1186/s12874-020-0897-3 31931747 PMC6958795

[49] Langdon J, Schlote R, Harris B, Burdette G, Rothberger S. Effects of a training program to enhance autonomy supportive behaviors among youth soccer coaches. JHSE. 2015;10(1):1. doi: 10.14198/jhse.2015.101.01

[50] Larsen T, Van Hoye A, Tjomsland HE, Holsen I, Wold B, Heuzé J-P, et al. Creating a supportive environment among youth football players. Health Educ. 2015;115(6):570–86. doi: 10.1108/he-04-2014-0054

[51] Strachan L, MacDonald DJ, Côté J. Project SCORE! Coaches’ perceptions of an online tool to promote positive youth development in sport. Int J Sports Sci Coach. 2016;11(1):108–15. doi: 10.1177/1747954115624827

[52] Søvik ML, Larsen T, Tjomsland HE, Samdal O, Wold B. Barriers in Implementing Coach Education in Grassroots Youth Football in Norway. Int Sport Coach J. 2017;4(2):162–76. doi: 10.1123/iscj.2016-0106

[53] Solstad BE, Larsen TMB, Holsen I, Ivarsson A, Ronglan LT, Ommundsen Y. Pre- to post-season differences in empowering and disempowering behaviours among youth football coaches: a sequential mixed-methods study. Sports Coach Rev. 2018;7(2):113–41. doi: 10.1080/21640629.2017.1361166

[54] Cope E, Cushion CJ, Harvey S, Partington M. Investigating the impact of a Freirean informed coach education programme. Phys Educ Sport Pedagogy. 2021;26(1):65–78. doi: 10.1080/17408989.2020.1800619

[55] Barrero AM, Abad Robles MT, Giménez Fuentes-Guerra FJ. Profile of grassroots football coaches of Spanish professional clubs. Kinesiology (Zagreb, Online). 2022;54(2):325–34. doi: 10.26582/k.54.2.14

[56] O’Brien W, Hogan I, Coppinger T. Coaches’ Experience of the “Gaelic4Teens” Program in Ireland. Int Sport Coach J. 2022;10(1):70–7. doi: 10.1123/iscj.2021-0094

[57] Barnes CS, Curtner-Smith MD. “From a Learning Perspective, It’s a Better Way for Them to Learn”: Impact of an Education Program on Two Youth Soccer Coaches’ Perspectives and Practices. J Teach Phys Educ. 2023;43(1):62–71. doi: 10.1123/jtpe.2022-0100

[58] Barnes CS, Curtner-Smith MD. “Maybe I’ll get some … new activities”: influence of an education programme on one grassroots youth soccer coach. Sports Coach Rev. 2024;1–24. doi: 10.1080/21640629.2024.2417146

[59] Swartz HS, Dieffenbach K, Swisher A. Creating community to support youth sport coach development. Quest. 2025;1–22.

[60] Peters MDJ, Godfrey CM, McInerney P, Munn Z, Tricco AC, Khalil H. Scoping Reviews (2020). In: Aromataris E, Lockwood C, Porritt K, Pilla B, Jordan Z, editors. JBI Manual for Evidence Synthesis. JBI; 2024.

[61] Braun V, Clarke V. Thematic analysis: a practical guide. Sage; 2022.

[62] Varghese M, Ruparell S, LaBella C. Youth Athlete Development Models: A Narrative Review. Sports Health. 2022;14(1):20–9. doi: 10.1177/19417381211055396 34758649 PMC8669922

[63] Holt NL, Neely KC, Slater LG, Camiré M, Côté J, Fraser-Thomas J, et al. A grounded theory of positive youth development through sport based on results from a qualitative meta-study. Int Rev Sport Exerc Psychol. 2017;10(1):1–49. doi: 10.1080/1750984X.2016.1180704 27695511 PMC5020349

[64] Vierimaa M, Turnnidge J, Bruner M, Côté J. Just for the fun of it: coaches’ perceptions of an exemplary community youth sport program. Phys Educ Sport Pedagogy. 2017;22(6):603–17. doi: 10.1080/17408989.2017.1341473

[65] Lyle J, Crespo M. Global coach development in a knowledge society: Critical issues. 1 ed. United Kingdom: Routledge; 2024. p. 357–72.

[66] Shaikh M, Santos YYS d, Rodrigue F, Ciampolini V, Galatti LR, Seguin C, et al. A scoping review of sport coach education programs: An international perspective. 2019.

[67] O’Gorman J. Introduction: developing the research agenda in junior and youth grassroots football culture. Soccer Soc. 2016;17(6):793–9.

[68] Brazier R, Lara-Bercial S, Hill M, Hodgson G, Megicks BS. The UK Youth Sport Coaching Workforce Report. Int Sport Coach J. 2026;13(2):259–67.

[69] Nash CS, Sproule J, Horton P. Excellence in coaching: The art and skill of elite practitioners. Res Quart Exerc Sport. 2011;82(2):229–38. 10.1080/02701367.2011.10599750 21699102

[70] Stodter A, Cushion CJ. Evidencing the impact of coaches’ learning: Changes in coaching knowledge and practice over time. J Sports Sci. 2019;37(18):2086–93. doi: 10.1080/02640414.2019.1621045 31135293

[71] Nelson LJ, Shulman D, Potrac PA, Gale LA, Ives BA. Maintaining professional face: deceptive impression management in community sport coaching. Sport Educ Soc. 2025;30(6):768–82. doi: 10.1080/13573322.2024.2349954

[72] Cushion CJ, Armour KM, Jones RL. Coach Education and Continuing Professional Development: Experience and Learning to Coach. Quest. 2003;55(3):215–30. doi: 10.1080/00336297.2003.10491800

[73] Campbell S, Waller S. Systematic Review of the Contemporary Research on Coach Development Programs. Int J Coach Sci. 2020;14(1).

[74] Nelson LJ, Potrac P, Groom R, Nelson L, Groom R, Potrac P. Recognizing the dimensions and tensions of learning in coaching. 1 ed. Routledge; 2016. p. 227–37.

[75] Ciampolini V, Milistetd M, Rynne SB, Brasil VZ, do Nascimento JV. Research review on coaches’ perceptions regarding the teaching strategies experienced in coach education programs. Int J Sports Sci Coach. 2019;14(2):216–28. doi: 10.1177/1747954119833597

[76] Trudel P, Gilbert W, Werthner P. Coach education effectiveness. In: Lyle J, Cushion C, editors. Sport coaching: Professionalisation and practice. Elsevier; 2010. p. 135–52.

[77] Cushion CJ, Nelson L, Armour K, Lyle J, Jones R, Sandford R, et al. Coach learning and development: a review of literature. 2010.

[78] Lyle J. Sports coaching concepts: A framework for coaches’ behaviour. Routledge; 2002.

[79] Driska AP, Nalepa J. Self-paced online learning to develop novice, entry-level, and volunteer coaches. Coach education and development in sport. Routledge; 2019. p. 166–77.

[80] Weiss MR. Youth Sport Motivation and Participation: Paradigms, Perspectives, and Practicalities. Kinesiol Rev. 2019;8(3):162–70. doi: 10.1123/kr.2019-0014

[81] Harvey S, Light RL. Questioning for learning in game-based approaches to teaching and coaching. Asia-Pac J Health Sport Phys Educ. 2015;6(2):175–90. doi: 10.1080/18377122.2015.1051268

[82] Griffiths MA, Armour KM, Cushion CJ. ‘Trying to get our message across’: successes and challenges in an evidence-based professional development programme for sport coaches. Sport Educ Soc. 2018;23(3):283–95. doi: 10.1080/13573322.2016.1182014

[83] Paquette K, Trudel P. The Evolution and Learner-Centered Status of a Coach Education Program. Int Sport Coach J. 2018;5(1):24–36. doi: 10.1123/iscj.2017-0038

[84] McQuade S, Nash C. The Role of the Coach Developer in Supporting and Guiding Coach Learning. Int Sport Coach J. 2015;2(3):339–46. doi: 10.1123/iscj.2015-0059

[85] Webb K, Leeder TM. Dispositions and coaching theories: understanding the impact of coach education on novice coaches’ learning. Sport Educ Soc. 2022;27(5):618–31. doi: 10.1080/13573322.2021.1887846

[86] Dempsey N, Cope E, Richardson DJ, Littlewood MA, Cronin CJ. Less may be more: how do coach developers reproduce “learner-centred” policy in practice? Sports Coach Rev. 2021;10(2):203–24.

[87] Lave J, Wegner E. Situated learning: legitimate peripheral participation. Cambridge University Press; 1991.

[88] Wenger E. Communities of practice: learning, meaning, and identity. Cambridge: Cambridge University Press; 1998.

[89] Cushion CJ, Stodter A, Clarke NJ. ‘It’s an experiential thing’: the discursive construction of learning in high-performance coach education. Sport Educ Soc. 2022;27(7):844–61. doi: 10.1080/13573322.2021.1924143

