# Correction: Youth participation coach learning: A scoping review

**DOI:** 10.1371/journal.pone.0359524

**Published:** 2026-09-29

**Authors:** The PLOS One Staff

Table 2 was uploaded incorrectly. Please see the correct Table 2 here.

The publisher apologizes for the error.

**Table 2:** Characteristics of included studies.

General study details			Coach Participants				Methodology			
Author (year)	Country of study	Sport type	Gender (*N*)	Coaches' experience	Prior coaching qualifications (*N*)	Coaches' Status	Study aim	Study design	Data collection	Theoretical construct
Langdon et al. (2015) [51]	USA	Team: Football	M = 5; F = 2	Mean = 6.43 yrs (SD = 6.44) (Intermediate)	NS	V	To evaluate the prevalence and implementation of a training emphasizing the use of autonomy supportive coaching behaviours among youth soccer coaches in game-play situations as well as evaluating its effects on motivational processes among athletes.	LONG: Mixed-methods explanatory research	QUAN: Athlete questionnaire; Coaching behaviours * QUAL: Coaches' reflections (during CEP)	Self-determination theory
Larsen et al. (2015) [52]	France Norway	Team: Football	FRA: M = 5; F = 1 NOR: M = 10; F = 2	FRA: Mean = 5.67 yrs (SD = 1.63) NOR: Mean = 6.92 yrs (SD = 5.26) (Intermediate)	Yes = 9; No = 9	V	To examine how grassroots coaches in youth football perceive their coaching practices after participating in a community-based coach education program aimed at optimizing their experiences in youth sport, namely the Empowering Coaching Training Program, based on self-determination theory and achievement goal theory.	CS: Qualitative exploratory research	QUAL: 1-1 interview (post-CEP)	Self-determination theory; achievement goal theory
Strachan et al. (2016) [53]	Canada	Team: Football, Hockey, Volleyball	M = 3; F = 1	4 to 12 yrs (*N* = 4) (Mixed)	NS	NS	To design and deliver a technology-based Positive Youth Development program (Project SCORE!) and explore its impact on coaches.	CS: Qualitative exploratory research	QUAL: 1-1 interview (post-CEP)	Positive youth development; ecological theory
Søvik et al. (2017) [54]	Norway	Team: Football	QUEST: M = 102; F = 11 INT: M = 10; F = 2	QUEST: Mean = 7.01 yrs (SD = 4.82) INT: Mean = 6.92 yrs (SD = 5.26) (Intermediate)	QUEST: NS INT: Yes = 5; No = 7	NS	To explore grassroots coaches’ perceptions of the content of a one-day coach education workshop (Empowering Coaching Training Program), the program’s applicability, their use of the content, and the perceived barriers to implementing the program in their coaching practice.	CS: Convergent parallel mixed-methods exploratory research	QUAN: Questionnaire (post-CEP) QUAL: 1-1 interview (post-CEP)	Effective program implementation
Solstad et al. (2018) [55]	Norway	Team: Football	QUEST: M = 174; F = 19 * * INT: M = 10; F = 2	QUEST: Mean = 4.26 yrs (SD = 2.10) * * INT: Mean = 6.92 yrs (SD = 5.26) (Intermediate)	QUEST: Yes = 106; UN = 87 * * INT: NS	NS	To investigate differences in self-reported behaviours among youth football coaches from pre- to post-season, and additionally, their evaluative reactions to participation in the Norwegian arm of the Empowering Coaching Training Program.	LONG: Sequential mixed-methods explanatory research	QUAN: Questionnaire (pre- and post-season) * * QUAL: 1-1 interview (post-season)	Self-determination theory; achievement goal theory
Cope et al. (2021) [56]	England	Mixed	M = 1; F = 2	3 to 6 yrs (*N* = 3) (Intermediate)	Yes = 3	Mixed	To investigate the impact a theoretically informed learning programme had on coaches’ ownership of, and feelings towards, being engaged in their learning, and how this impacted their subsequent practice.	LONG: Mixed-methods explanatory research	QUAN: Coaching observation (during CEP) * QUAL: 1-1 interview (pre- and post-CEP); reflective conversations (during CEP)	Critical pedagogy; collaborative action research
Barrero et al. (2022) [57]	Spain	Team: Football	M = 140; F = 13	4 to 7 yrs (*N* = 61) 7 to 10 yrs (*N* = 44) > 10 yrs (*N* = 14) (Mixed)	Yes (Level 2) = 71; Yes (Level 3) = 60 Some NS	NS	To identify the profile and education of grassroots football coaches (working with 8- to 12-year-olds) of Spanish professional clubs (first and second male division, and first female division), as well as the qualities and characteristics they should have.	CS: Quantitative exploratory research	QUAN: Questionnaire	NS
O’Brien et al. (2022) [58]	Ireland	Team: Gaelic Football	M = 5; F = 3	2 to 20 yrs (*N* = 5) UN (*N* = 3) (Mixed)	Yes = 6; UN = 2	V	To examine Irish volunteer coaches’ experiences of the content and delivery of the Gaelic4Teens coach education initiative and further seeks to evaluate if participants coaching behaviour changed as a result.	LONG: Qualitative explanatory research	QUAL: Focus-group interview (pre- and post-CEP)	NS
Barnes and Curtner-Smith (2023) [59]	USA	Team: Football	M = 2	6 yrs and 10 yrs (Intermediate)	No = 2	V	To describe the impact of a progressive coach education program on two grassroots youth soccer coaches’ perspectives and practices, and the factors that helped and hindered the CEP’s effectiveness.	LONG: Mixed-methods descriptive case study	QUAN: Coaching observation (pre- and post-CEP) * QUAL: 1-1 interview (pre-, during, and post-CEP); coach-created materials (pre- and post-CEP)	Occupational socialization theory
Barnes and Curtner-Smith (2024) [60]	USA	Team: Football	M = 1	About 3 yrs (Novice)	No = 1	V	To describe how one grassroots youth soccer coach was influenced by a progressive coach education programme, and the factors that supported or negated the CEP’s effectiveness.	LONG: Mixed-methods descriptive case study	QUAN: Coaching observation (pre- and post-CEP) * QUAL: 1-1 interview (pre-, during, and post-CEP); coach-created materials (pre- and post-CEP)	Occupational socialization theory
Swartz et al. (2025) [61]	USA	Mixed	P1: M = 17; F = 16 P2: M = 18; F = 24	P1: Mean = 4.72 yrs (SD = 5.47) P2: Mean = 6.14 yrs (SD = 4.44) (Intermediate)	NS	V	To explore the impact of the Youth Sport Coach Fellowship Program which was aimed at empowering grassroots coaches with the knowledge, resources, and networks needed to promote holistic athlete development and foster positive youth outcomes both on and off the field.	LONG: Mixed-methods exploratory action research	MIXED: Written and video assignments, discussion posts, reflections (during CEP); survey (post-CEP)	Adult learning theory

CEP Characteristics					Key findings
Learning source (mode)	Name of CEP	CEP scale (duration)	Key content / topics	Key delivery / strategies	
Formal (Both)	"Autonomy-supportive coaching training"	SM (12 weeks)	Autonomy-supportive coaching	Written and verbal feedback by coach developer; supplementary online learning; systematic observation of coaching behaviours; self-reflection	Coaches were not able to significantly modify their coaching behaviours in game play yet reflectively reported modest implementation of autonomy supportive behaviours. Coaching experience influenced the variety of autonomy supportive coaching behaviours demonstrated. Coaches believed the training influenced their coaching style/philosophy in relation to coach-athlete relationship and communication styles, emphasizing choice and rationales.
Formal (Both)	Empowering Coaching Training Program	SM (1 day)	Autonomy-supportive coaching	Coach developer-facilitated workshop; supplementary workbook and online learning	All coaches had a shared understanding of the philosophy of the CEP, and to apply several of the strategies they had learnt during the workshop. The coaches perceived that the CEP supported their efforts to develop and implement strategies to stimulate intrinsic motivation, enjoyment and long-term participation among the players. There were some differences between coaches from France and Norway (e.g., rules and involvement), but the similarities were more evident, supporting the universality of applying self-determination theory in the youth sport setting.
Informal (Online)	Project SCORE!	SM (6 to 12 weeks)	Holistic youth coaching (Positive Youth Development)	Self-paced online learning	Four themes (and sub-themes) were reported in the pilot implementation: (a) program organization (preparation, delivery of lessons); (b) positive aspects about the program (website, lessons/sessions, personal growth, parent reaction); (c) challenges about the program (participant involvement, time away from training); and (d) suggestions for change (mix and match lessons, applicability to younger ages).
Formal (Both)	Empowering Coaching Training Program	SM (1 day)	Autonomy-supportive coaching	Coach developer-facilitated workshop; supplementary online learning	The quantitative results indicate that the coaches were mainly positive about the CEP content and found it easy to apply and adapt to. Few coaches seemed to apply the program principles to a great extent. Qualitatively, coaches reported barriers related to program characteristics (i.e., lack of follow-up); individual factors (i.e., lack of time; players' time); and organizational factors (i.e., lack of support from club leadership or club officials, lack of a shared understanding of the program with their co-coaches).
Formal (Both)	Empowering Coaching Training Program	SM (1 day)	Autonomy-supportive coaching	Coach developer-facilitated workshop; supplementary coaching manual and online learning	Coaches’ empowering and disempowering behaviours did not differ from pre- to postseason. Qualitatively, the post-season interviews showed that participation in the CEP led coaches to reflect on their coaching practices, facilitating an increased focus on enabling autonomy and involvement for the athletes and more attention paid to the athletes’ feelings of mastery.
Formal (Both)	"Freirean-informed coach education program"	SM (9 to 13 weeks)	Coaching practice; Coach learning	Online resources (blogs, podcasts and videos); coach developer-facilitated reflective conversations; systematic coaching observations	Based on Freire's critical pedagogy, three themes were identified based on analysis: (a) freedom to learn, (b) feeling cared for, and (c) becoming (or not becoming) more reflexive. Quantitative data highlighted where this led to changes in the coaches' behaviours.
NA	NA	NA	NA	NA	Coaches considered it very important to have pedagogical skills and to use coaching methods that focus on learning rather than on results. Coach participants considered that the training received in the official coaching courses did not adequately qualify them to carry out the sporting and human education of boys and girls in the under-10 and under-12 age groups. The coaches also felt that the contents of the coaching course should be adjusted to the requirements of these age groups.
Formal (Both)	Gaelic4Teens Program	SM (16 weeks)	Autonomy-supportive coaching and National Sport Federation guidelines	Face-to-face session; live webinars; self-paced online learning; class discussion via e-learning platform	Overall, the CEP had a meaningful impact on coaches' abilities to competently work with female adolescents. Three overarching themes were: (a) coach education: coach appreciation of online and blended learning approaches; (b) coach environment: the coach’s provision of player autonomy, conﬁdence and competence; and (c) coach behaviour: holistic coaching philosophy, using robust coaching pedagogies in practice.
Formal (Both)	United States Soccer Federation Grassroots Coaching License	LG (1 day)	Coaching pedagogy (Game-based coaching)	Introductory online module; coach developer-facilitated classroom discussion; session planning with on-field coaching; systematic observations of coaching behaviours	The CEP had a significant impact on one coach but negligible on the other coach. The two coaches’ occupational socialization helped explain these differential effects. The study suggests that CEPs should have a greater impact on coaches if they are relatively lengthy, include follow-up support, and coach developers are aware of coaches’ acculturation and organizational socialization
Formal (Both)	United States Soccer Federation Grassroots Coaching License	LG (1 day)	Coaching pedagogy (Game-based coaching)	Introductory online module; coach developer-facilitated classroom discussion; session planning with on-field coaching; systematic observations of coaching behaviours	The CEP had little influence on the coach's beliefs and pedagogies. The coach’s professional socialization was not powerful enough to overcome his acculturation or his organizational socialization. The study indicates the need for lengthy CEPs, follow-up support and suggests coach developers should deconstruct their coaches’ acculturation and mediate any negative organizational socialization to which coaches may be subjected.
Formal (Online)	Youth Sport Coach Fellowship Program	SM (Phase 1: 26 weeks, Phase 2: 14 weeks)	Holistic youth coaching	Online synchronous learning (e.g., live class discussion, coach developer-led video calls) and asynchronous learning (e.g., self-paced learning modules, capstone project)	Overall, the action research approach supported exploring the design of the CEP in terms of its effectiveness on coaches. Four key areas in relation to the study purpose were: (a) combating coaching isolation; (b) supporting coaches' desire to learning; (c) harness the power of cross-sport connections; and (d) importance of well-prepared facilitation.

*Only the qualitative component of this study met the inclusion criteria and was included in the analysis; the quantitative component was excluded.

**Only the qualitative sample of this study met the inclusion criteria and was included in the analysis; the quantitative component was excluded.

Note. M = male; F = female; FRA = France; NOR = Norway; P1 = Phase 1; P2 = Phase 2; UN = Unknown; NS = Not stated in study; UEFA = Union of European Football Associations; FT = Full-time; PT = Part-time; V = Volunteer; QUEST: Questionnaire; INT: Interview; CS = Cross-sectional; LONG = Longitudinal; QUAN = Quantitative; QUAL = Qualitative; MIXED = Mixed-methods; NA = Not Applicable; SM = Small-scale; LG = Large-scale.
