# Supplementary material for: Youth participation coach learning: A scoping review
Source: PLoS One. 2026 Aug 18;21(8):e0356171. doi: 10.1371/journal.pone.0356171 (PMC13485061; doi:10.1371/journal.pone.0356171)
Supplement: Supplementary file 2 [file pone.0356171.s002.docx]

#### S3 Table. Study context and participant characteristics

| **Country of study** | **No** | **%** |
| --- | --- | --- |
| England | 1 | 9.1% |
| France and Norway | 1 | 9.1% |
| Ireland | 1 | 9.1% |
| Norway | 2 | 18.2% |
| Spain | 1 | 9.1% |
| Europe | 6 | 54.5% |
| Canada | 1 | 9.1% |
| USA | 4 | 36.4% |
| North America | 5 | 45.5% |
| **Sport** | **No** | **%** |
| Gaelic Football | 1 | 9.1% |
| Football | 7 | 63.6% |
| Football, Hockey, Volleyball | 1 | 9.1% |
| Team | 9 | 81.8% |
| Individual | 0 | 0.0% |
| Mixed (Team/Individual) | 2 | 18.2% |
| **Gender (*N*)** | **No** | **%** |
| Males only | 2 | 18.2% |
| Females only | 0 | 0.0% |
| Mixed | 9 | 81.8% |
| ***Coaches' experience** | **No** | **%** |
| Novice (<3 yrs) | 1 | 9.1% |
| Intermediate (3-10yrs) | 7 | 63.6% |
| Experienced (10+ yrs) | 0 | 0.0% |
| Mixed | 3 | 27.3% |
| **Prior coaching qualifications (*N*)** | **No** | **%** |
| Yes | 1 | 9.1% |
| No | 2 | 18.2% |
| Mixed (Yes and No / Not stated or Unknown) | 4 | 36.4% |
| Not stated | 4 | 36.4% |
| **Coaches' status** | **No** | **%** |
| FT only | 0 | 0.0% |
| PT only | 0 | 0.0% |
| V only | 6 | 54.5% |
| Mixed | 1 | 9.1% |
| Not stated | 4 | 36.4% |

*Based on mean or range of overall population
