# Supplementary material for: Youth participation coach learning: A scoping review
Source: PLoS One. 2026 Aug 18;21(8):e0356171. doi: 10.1371/journal.pone.0356171 (PMC13485061; doi:10.1371/journal.pone.0356171)
Supplement: Supplementary file 3 [file pone.0356171.s003.docx]

#### S4 Table. Coach Education Program (CEP) characteristics

| **Source of learning** | **N** | **%** |
| --- | --- | --- |
| Formal | 9 | 81.8% |
| Informal | 1 | 9.1% |
| Not applicable | 1 | 9.1% |
| **Mode of learning** | **N** | **%** |
| In-person | 0 | 0.0% |
| Online | 2 | 18.2% |
| Both | 8 | 72.7% |
| Not applicable | 1 | 9.1% |
| **CEP scale** | **N** | **%** |
| Large | 2 | 18.2% |
| Small | 8 | 72.7% |
| Not applicable | 1 | 9.1% |
| ***CEP maximum duration** | **N** | **%** |
| < 1 week | 5 | 45.5% |
| 1 week to 12 weeks | 2 | 18.2% |
| > 12 weeks | 3 | 27.3% |
| Not applicable | 1 | 9.1% |
| **CEP key content / topics** | **N** | **%** |
| Autonomy-supportive coaching | 5 | 45.5% |
| Coaching pedagogy | 2 | 18.2% |
| Holistic youth coaching | 2 | 18.2% |
| Others / general coaching-related | 1 | 9.1% |
| Not applicable | 1 | 9.1% |

*Some CEPs ranged in duration
